# Extreme Contagiousness of Scarlet Fever

**Published:** 1871-02

**Authors:** 


Extreme Contagiousness of Scarlet Fever.—A distinguished British physician, writing in the London *Lancet,* and advocating the contagiousness of scarlet fever, mentions the case of a man who was seized with the disease after opening and reading a letter from a friend affected with it, and who wrote: "Even while I write you, my hands are skinning." It is not stated positively that the disease came in the letter, but such is the inference. We in California have heard of still more astonishing results from letters written by men on this coast to their wives in the Atlantic States.—*Ibid.*

